# P08-14 The association of childhood commuting modes and physical activity in adult age

**DOI:** 10.1093/eurpub/ckac095.127

**Published:** 2022-08-29

**Authors:** Kaisa Kaseva, Tuija Tammelin, Xiaolin Yang, Janne Kulmala, Harto Hakonen, Olli Raitakari, Kasper Salin

**Affiliations:** 1 Faculty of Sport and Health Sciences, University of Jyväskylä, Jyväskylä, Finland; 2 LIKES Research Centre for Physical Activity and Health, University of Jyväskylä, Jyväskylä, Finland; 3 LIKES Research Centre for Physical Activity and Health, Jyväskylä, Finland; 4 Department of Clinical Physiology and Nuclear Medicine, Turku University Hospital, -, Turku, Finland

**Keywords:** active commuting, self-reported physical activity, accelometer-measured physical activity, longitudinal study

## Abstract

**Background:**

Physically active lifestyle prevents and contributes to managing non-communicable diseases. Childhood physical activities have shown to associate with physically active lifestyle in adulthood. More research on which childhood physical activity modes associate with physical activity in later life is still needed. Within the present study, we examined how physically active commuting to school in childhood contributed to overall physical activity in adulhood.

**Methods:**

The participants (N = 3596) were from the population-based, longitudinal Cardiovascular Risks in Young Finns Study. Questionnaires were used in assessing subjects' childhood (1980) and adulthood (2001-2018) physical activity. ActiGraph accelerometers were also applied in the adulthood measurements (2018-2020). The results were analyzed using logistic and linear regression models. Participants' age, sex, parents' educational background, parents' income level, childhood living area, participants' educational background, adulthood income level, and adulthood living area were adjusted for in the models.

**Results:**

Based on the preliminary examinations, childhood commuting was not associated with self-reported commuting to work (2001-2018) or accelerometer-measured overall physical activity (2018-2020) in adulthood (p>.05). Active commuting in childhood associated with increased self-reported leisure-time physical activity in the year 2001 (b=.38, p>.001), 2007 (b=.35, p>.001), and 2018 (b=.28, p=.012), but the association between childhood commuting and self-reported physical activity in the years 2001 and 2018 attenuated after adjusting for all covariates (p>.05).

**Conclusions:**

Physically active commuting in childhood (1980) was associated with higher levels of self-reported leisure-time physical activity in adulthood (2001-2018). The associations attenuated after adjusting for covariates excluding the one between active commuting and leisure-time physical activity assessed in 2007. Physically active commuting can be regarded as recommendable with respect to the development of physically active lifestyle, if supportive evidence for the causality between childhood commuting and leisure-time physical activity in adult age can be found. Future research should also focus on assessing whether active commuting in childhood contributes to adulthood activities parallel to active commuting in childhood.

